# Online help for people with suicidal thoughts provided by charities and healthcare organisations: a qualitative study of users’ perceptions

**DOI:** 10.1007/s00127-020-01852-6

**Published:** 2020-03-10

**Authors:** Lucy Biddle, Jane Derges, Carlie Goldsmith, Jenny L. Donovan, David Gunnell

**Affiliations:** 1 Population Health Sciences, Bristol Medical School, University of Bristol, Canynge Hall, 39 Whatley Road, Bristol, BS8 2PS UK; 2 The National Institute for Health Research Applied Research Collaboration West (NIHR ARC West) at University Hospitals Bristol NHS Foundation Trust, Bristol, UK; 3 Samaritans, Ewell, Surrey UK; 4 National Institute for Health Research Bristol Biomedical Research Centre, University Hospitals Bristol NHS Foundation Trust and University of Bristol, Bristol, UK

**Keywords:** Suicide, Self-harm, Help-seeking, Internet, Qualitative research

## Abstract

**Purpose:**

Internet use is common among people with suicidal feelings and a considerable amount of suicide help material is available online. Despite attempts to promote formal help sites (e.g. governmental and charity sector) in internet search results, users’ evaluation of these sites is lacking. This study, therefore, aimed to explore distressed users’ perceptions of formal online help and their experiences of using this in times of crisis.

**Methods:**

In-depth interview study of 53 adults reporting suicide-related internet use.

**Results:**

While highly valued in relation to general mental health problems, formal sites were not perceived to meet the different needs of those experiencing suicidal thoughts, and did not engage individuals in crisis. Sites were criticised for being impersonal, dispassionate, too focused on information-giving, and lacking solutions that were novel or sensitive to reasons why an individual may choose to seek help online. Most participants criticised the tendency for sites to signpost to offline services as their primary response. Participants desired immediacy and responsive online help incorporating ‘live chat’, self-help tools, opportunities to interact with others and lived-experience content. Positive accounts of seeking online help described sites incorporating these features.

**Conclusions:**

Formal online help services should be reappraised to ensure they meet users’ needs for immediacy and responsive help to capitalise upon the opportunity available for suicide prevention.

## Introduction

Suicide-related internet use is common in people with suicidal feelings, particularly as severity of ideation increases [1]. In a recent study [2], 45% of young people with suicidal thoughts and 70% who had made a suicide attempt reported suicide-related internet use. Within a hospital emergency department setting, 8% of adults who had seriously self-harmed reported suicide-related internet use in relation to their presenting episode. This rose to one in four amongst those with high suicidal intent and was more frequent in young people [3]. While such use may be harmful, it is also recognised that the online world can offer a powerful tool for suicide prevention [4, 5].

Internet search studies indicate a considerable amount of suicide help material is available online [6]. While this may not always (or even commonly) be sought by distressed users [7], search algorithms, language triggers, and other devices, mean links to help pages frequently appear prominently in search results or as ‘pop ups’ where suicide-related terms are entered into search engines. For instance, in the UK, Samaritans (a suicide prevention charity) has collaborated with Google to ensure a box advertising their services is displayed at the top of such searches.

To date, research has not explored users’ perceptions of online suicide help and emotional support offered by official healthcare and mental health/ suicide prevention charity sector websites (hereafter ‘formal’). This is the aim of this paper. We conducted qualitative interviews with over 50 individuals who used the internet when experiencing suicidal ideation.

## Methods

In-depth interviews were carried out within a large-scale study of suicide-related internet use, exploring risks and benefits of use. As part of this, participants were asked to describe their experiences of the online world as a source of help when feeling suicidal/distressed.

### Sampling

Methods are detailed elsewhere [7]. Briefly, interviews were carried out between 2014 and 2016. Participants were included if they were English speaking, had experienced suicidal thoughts/behaviour and reported internet use in relation to these—‘suicide-related internet use’. Participants were purposively sampled from:

1. Sample YP—community-based sample of young people (21–23 years) from The Avon Longitudinal Study of Parents and Children (ALSPAC cohort) [8] reporting suicide-related internet use in a questionnaire [2];
2. Sample SH—hospital patients (18+ years) presenting to the Emergency Departments of two major hospitals in South West England following a suicide attempt and reporting suicide-related internet use at psychosocial assessment [3];
3. Sample SM—community-based sample of adults (18+ years) reporting suicide-related internet use in an online survey by Samaritans, a prominent charity supporting suicidal individuals in the UK and Republic of Ireland.

Multiple samples ensured diversity and that internet use at differing stages across the suicidal trajectory could be explored. Sample YP included some individuals reporting lower levels of distress and also individuals not in contact with services: less than half had sought medical help and three reported no support seeking at all (including school counselling or voluntary services). Sample SH were recruited both prospectively (while in hospital) and retrospectively (attempt within past year) following a search of assessment proformas, and thus included individuals recently displaying high suicidal intent. Patients were invited if deemed well enough by the inviting clinician and able to give informed consent. Sample SM helped to fill gaps arising from other samples by recruiting participants from ethnic minorities (Asian, *n* = 2; Dual Heritage, *n* = 3) and of older age, including two participants older than 55 years. Individuals in samples YP and SM were eligible if reporting life-time suicidal thoughts and suicide-related internet use. Recruitment continued until a wide range of participants had been interviewed, including ‘deviant cases’ [9], and consistent findings were emerging.

### Data collection

Interviews were conducted by JDe (Samples YP and SH) and CG (Sample SM). Most were face-to-face but nine were conducted by telephone, due to geographical spread. All were audio recorded. Most lasted between 1 and 2 h. All participants gave informed consent. Data collection and analysis occurred simultaneously to ensure understanding was incremental. An interpretative approach was used in which participants were encouraged to talk at length, in their own terms, identifying the issues they considered of importance to the topic and providing detailed narratives of their internet use and the meanings it held for them in relation to their suicidal feelings/behaviour. Probing was used to mine salient points and encourage reflection. A topic guide promoted consistency between researchers and ensured the core issues were explored with each participant. This was used flexibly according to the direction taken by participants and was revised to incorporate emerging themes. Regular meetings were held between interviewers to discuss ‘data leads’. Online help-seeking and evaluation of help sites were included in the topic guide and covered with all participants, even if not arising spontaneously. Two participants who had not seen help content were shown materials and asked for their views, though analysis prioritised actual experiences. The research steering group included two advisors with lived-experience of suicidal thoughts and internet use, who contributed to the initial topic guide and commented on emerging findings.

### Analysis

Verbatim transcripts were coded to identify key themes. A third were independently double coded to check reliability. Initially, samples were analysed separately, led by a different researcher (JDe: YP; LB: SH, CG: SM), but for comparison, attempts were made to assimilate coding frames where possible and use consistent terminology. Codes were amalgamated into higher order concepts, or sub-divided as a more refined understanding emerged. All data relating to help use were retrieved and analysis progressed using constant comparison [9], with data being compared within and across individuals and samples to identify similarities and differences and how these could be accounted for. This included a focus on ‘deviant cases’ and exploring themes across longitudinal narratives and according to levels of suicidal intent. NVivo v.10 was used. Quotations are presented below tagged with sample and participant number.

## Results

Fifty-three individuals were interviewed (YP = 13; SH = 20; SM = 20) (Table 1). There were 31 females and 22 males. Ages ranged from 19 to 69 years. While relaying their narratives, all participants self-reported episodes of suicidal thoughts or self-harm behaviour, most (*n* = 34) described a suicide attempt, and many reported having a psychiatric problem or formal diagnosis. Sample SH appeared to describe the most severe attempts, though formal measurement of suicidality or morbidity was not carried out in any sample. Almost all had been in contact with health services in relation to mental distress. A small number (mostly Sample SM) reported help-seeking from voluntary sector services.

**Table 1:** Participant characteristics

Characteristic	Community-based young people (YP) (*n* = 13)	Hospital patients (SH) (*n* = 20)	Community-based adults (SM) (*n* = 20)
Sex			
Male	6	8	8
Female	7	12	12
Age (years)			
18–34	13	13	8
35–54	–	7	10
55+	–	–	2
Range	22–24	19–51	21–69
Mean	23	31.6	^a^
Suicide attempts			
Unclear	2	0	0
None	8	1	8
1–3	3	11	4
3 or more	0	8	8
Self-reported life-time psychiatric disorder			
Yes	8	15	19
No	5	5	1

^a^Not available since for some participants, only age category was collected

### Patterns of online help-seeking

Many participants regularly used formal online help for on-going mental health difficulties. In this context, they evaluated sites positively as a ‘comprehensive’ (SH2), ‘accessible’ (SM109) information source for exploring diagnosis, treatments, and self-help advice—particularly in the early phases of mental illness. All but two participants had viewed suicide-related online help content. This included suicide-specific sites and suicide pages within general mental health sites but use of this in response to suicidal feelings/behaviour was less frequent and sustained.

Approximately, a fifth of participants had only viewed suicide help content indirectly rather than having deliberately sought this—for example, via a ‘pop up’ appearing during browsing around suicide or because they were told to access this by a clinician. Of the remaining participants, some deliberately searched for suicide-related help by entering search terms into a search engine, but most viewed it as an extension of their pre-existing use of general online mental health resources. Many participants described how their engagement with formal online suicide help had varied across their illness trajectory, typically occurring prior to the onset of suicidal intent (while feeling distressed but ambivalent about suicide) and/or after a crisis (e.g., a suicide attempt) when recovering or their ‘bad thoughts were dulled’ (SM107). When suicidal feelings were acute, most participants avoided or ignored help content on the basis they had decided to attempt suicide and did not want intervention, or considered they had ‘gone past [help]’ (SH18)/it was ‘too late (SH15)’. In this mindset, it was said looking at help content ‘doesn’t compute’ (SH2), and that pop-ups could seem ‘patronising’ (YP11).

[Internet searching] before [a suicide attempt] was predominantly information and support seeking and then in the immediate run-up it was more like details of how to [implement suicide] … there was a smattering of, ‘if you’re feeling suicidal’ kind of links that I just ignored because I was like, ‘well this isn’t what I’m looking for!’ … I was quite single-minded by that point… coming out of [suicide attempt] then I’d start to read [charity] websites. (SM35)

Thus, most described mixed feelings: a clear distinction being made between the use of formal help sites in the context of general mental health and their utility specifically when experiencing suicidal feelings or crisis:

I’ve been on [charity website] when I was confused about my diagnosis… I think they do a bloody good job of explaining things and making you feel less of an enigma to yourself… When it comes to suicide, I haven’t gone down that avenue… It certainly wouldn’t have been ‘oh, I want to look up suicide prevention’. I’ve never thought like that. (SH2)

Participants expressed differing help needs when suicidal, which led to a series of negative evaluations about formal online help offered in relation to suicidal feelings and behaviour. Themes were consistent across suicide-specific sites and suicide help pages within general mental health sites. Indeed, participants who had viewed content from both sources tended to talk about suicide help content in general terms. Differentiation is not, therefore, provided below.

### Negative evaluations of online help in the context of suicidal feelings

Negative evaluations occurred across samples and encompassed four main themes.

#### Impersonal care

Much formal online help was criticised for being impersonal, apparently emanating from ‘big organisations’ (SH15), and having a ‘corporate’ (SH2) appearance. Accordingly, it was perceived as dispassionate and unlikely to offer personal care or understanding. This sense was exacerbated where sites emphasised fundraising.

It’s almost like a shop window in a sense with these websites where actually they’ll go over what sort of work they’ve got, yes, they’ll have some contact details on there… but I think the fact of it on the internet is too much of a face, almost like a fascia… in terms of what it can help me with, what sort of personal help it can provide, I wouldn’t know… I don’t necessarily feel okay, this is my safe place. (SH2)

It needs to be information that isn’t, you know, ‘the train leaves at, arrives at’, because the user is looking for support, comfort and understanding, and so the dialogue, the actual way it’s presented is massively important because you know, putting in something like ‘suicide help me’, I’m not saying I’ve got toothache (SH17)

Participants argued sites frequently lacked ‘a sense of community’ or opportunity for ‘emotional connection’ (SM79). Consequently, they typically reported: ‘I would just read it rather than engaging with it’ (SM113). Contact with the site could feel remote or ‘like talking to a scarecrow’ (SH14) and may entail a significant wait before being answered:

I sent an online ‘I need help please’ [to charity]… 2 days later I got a reply. A very generic, ‘I’m sorry to hear you’re feeling this way’… I wouldn’t say they’re bad, just not something (pause), I know if I was ever struggling, I would use again (SH15).

#### Limitations of information-giving

While helpful for general mental health, information provision was considered insufficient in moments of crisis:

I don’t think it was like, ‘oh but I really wanted this information and it wasn’t there’. I think it was just I was in such a bad place that the information wasn’t enough (SM35).

However, an emphasis on information-giving was typical and could make sites appear ‘dry’ (YP1), ‘clinical’ (YP5), and inaccessible. Those visiting them commented, ‘I wouldn’t want to read through paragraphs and paragraphs’ (YP4). The information given was deemed ‘too basic’ (SM111), and not helpful at a point where participants needed responsive help or tools for recovery:

It will tell you what you already know: I know what suicide is, I know what self-harm is. And it’ll give you, ‘lots of people go through these things’—it’s a bit like grandad, ‘oh, you’ll be alright son’. And you think, I’m not in a position where I want to go ‘aah’. I’m in a position where I want to go ‘I need some [expletive] help here. I need some help now, right now’ (SH17)

#### Ill-fitting ‘solutions’ and the limitations of signposting

Where sites provided possible actions or self-help advice, these were mostly criticised for lacking uniqueness; that is, not being different to help provided offline:

I don’t want to be told you need to eat, you need to sleep, you need to see your GP because I’m doing all of that and well it’s not helping! I need something more (SM111).

They could also be perceived as insensitive or unrealistic when not tailored to the specific needs of individuals choosing to seek help online, such as those lacking social support, seeking anonymity, or fearing stigma:

A lot of sites say keep your friends close and make sure you talk to family… then you remember, ‘I don’t have any friends anymore because my mood swings have killed that’, my parents are just going to badger me, like you don’t really want your parents to know… it just makes you feel ‘well great, there’s no way of me actually helping myself’ (SH18)

Participants reported that most sites offer signposting as their primary line of response—typically offline to a general practitioner or telephone helpline. This was frequently regarded as the epitome of an ‘ill-fitting solution’ (detailed in Table 2). Participants were often already aware of the signposted services and some had contacted them previously. Indeed, some went online when external help-seeking seemed exhausted or unable to deliver the support they felt they needed. For those looking online to seek an alternative approach, signposting merely presented an ‘old’ or ‘ineffective’ solution and could convey the impression that help was not possible. Signposting also required an additional ‘step’ (SM113) that could feel too ‘hard’ (YP1) to pursue. For instance, many described themselves as uncomfortable, ‘too nervy’ (SH2), or as lacking the ‘privacy at home’ (SM33) to talk on the phone or face-to-face; some were unprepared to leave their home; and others lacked the motivation or energy to seek ‘real world’ help. These were all factors that had motivated a search online.

**Table 2:** Data extracts illustrating key themes relating to the limitations of signposting

Theme	Data extract
Old/‘ineffective’ solution	A lot of [sites] kind of, if you clicked in the seek help thing, it will say, ‘oh here’s the number for [charity]’, which I kind of had… and antidepressants and everything and just kind of like, ‘that should help’ but that’s help I’m already getting (SH6) The only solutions the internet give you is ‘go to a doctor’ and from my experience of going to doctors it doesn’t really help in the short-term. You just end up waiting months and months and months to find a solution (SH18) When it feels like you’re constantly getting back to the same places or constantly making the same phone calls it gets kind of a bit—you sort of start thinking that maybe this can’t be helped (YP13)
Barriers to following signposts	They don’t actually help you on the site, they help you find the help. And if people are feeling like they don’t want to live anymore, why would they make the effort then, once you’ve already made the effort to look for online help, why are you then going to do something else and pick up the phone… it’s so much effort when it’s easier to go the other way. (SH8) I think it’s so important to have self-help on the internet… it takes nothing to log onto the internet and have a look, but it took me all of my kind of courage and reserves and everything to ring the doctors. To actually go to the doctors. To get over my social anxiety, to go for therapy with somebody that I didn’t know. It is a big ask. (YP4)
Limited use in crisis	There’s no valid advice online telling you, ‘try this’… it’s all very well like ‘go and speak to your doctor’ but they are only open nine until five. That doesn’t help you at 10 o’clock that night when all you can think about is going down into the kitchen and getting a knife (YP1) When you actually read what they [NHS Choices] have to say, it’s signposting… not what I would consider to be crisis interventions. When you are two hours away from taking those pills, make an appointment with the GP, it’s not thought through (SH17)

Frustration arose where individuals clicked on a ‘get help’ link and merely found themselves signposted elsewhere. Several also drew attention to the limitations of signposting ‘out of hours’ or in a crisis where an instantaneous response is required. Essentially, participants wanted responsive help provided in situ (within the online environment in which they were looking).

The support is you can ‘phone or you can go in somewhere. But that’s about it (pause) there’s nothing else. There’s nothing online. And I think what I want is something instant, online. (SM79)

#### Lack of age-specific content

Some younger participants believed there was a lack of help provision aimed at young people, with contemporary role models, and providing ‘teenage-friendly self-help’ (YP9). However, some older participants argued that online material disproportionately focuses on young people.

### Suggestions for improvement and positive experiences of online help

Participants discussed how formal online help provision might be improved. Some also described help they had found useful (detailed in Table 3). Experiences and suggestions for improvement aligned.

**Table 3:** Data extracts illustrating key themes relating to positive experiences of online help

Theme	Data extract
Dialogue	Everyday [charity] post something and it’s a nice reminder that it’s going on still, so you don’t feel completely alienated and alone all the time… It’s almost like having a friend check up on you every day, the same logic. (SM109)
Links to moderated forums	[Site] had a banner saying if you need support now, click here, and then it kind of links you into the forums that you can join in and stuff. (Int: you feel that it was important that there was something immediately there?) Totally, yeah. I think if there hadn’t been, I don’t know what would have happened then. But yeah, no it was important. I mean there was people on-line typing… you could type a paragraph and then somebody would come back with the reply (SM1) When you sign up they came up with the rules of this site, which were very plainly put in a straightforward, talking sort of way. And then they put a little bit about each of the moderators and they’d got their pictures. Also, you could see where they’d intervene… you did feel as if there was a presence, and not an intimidating, policing presence, it would be more a hands on the tiller, if you want me, shout, sort of presence… that was very important, I thought. (SM33)
Lived-experience content	I liked that there was a balance between information given by professionals and then patient experience, which kind of humanised it I guess as well (SM35) You want something that makes you go [bangs on the table] that’s it—that’s the way I feel. (Int: And can you say what it was about that site that did that for you?) I read some stories and I didn’t see them as anecdotes. I saw them as a very real and very true… it gave me some courage… and it gives you the option to take onboard they [subjects] are still here and ultimately that’s extremely important because you can recognise yourself in what’s being said (SH17)
Self-help tools	The information didn’t change, it’s a static thing, I needed something extra then, something new or different. (Int: Can you recall anything that did feel different or new?) I think it was thinking to look for crisis plans, and I think it would have been better if they were more obviously accessible perhaps, rather than like I only found them because I thought to search for them… having something like that was very instructive… like step-by-step, and that then gave you something you could come back to at other times. (SM35)

#### Instant messaging and dialogue

‘Live chat’ or ‘instant messaging’ was frequently suggested as a way to transfer telephone helpline services into the online world and facilitate immediate access to a helper in a medium accessible for individuals who find direct talk too confronting or feel unable to follow signposting information:

All support organisations should have an online resource that people can actually access real people, not just a little message: ‘oh thank you for your message, we’ll get back to you in 24 hours’; you know, that’s useless. (SM1);

The reason I go online and look is those times when I’m alone, I’ve gone to bed, I know I’m not going to sleep … I don’t want to ring [helpline] because then you have to really talk to someone…and you don’t always want that, and I always think, ‘oh the neighbours would be able to hear me’… those times that I’m sat there with an iPad in my hands, and I just want (sighs) I just wish there was somebody there for me…for there to be an instant response (sighs), to be able to contact somebody—straight away—without having to talk to them. Because talking can be hard (SM79)

Some noted ‘live chat’ provided by formal help providers would be particularly preferable as there would be assurance that the responder was not only trained/‘a professional’ (SH9) but also resilient and ‘not vulnerable’ (SM35), which may not be the case with peer-to-peer communication.

One participant praised a charity’s use of social media to generate on-going dialogue and reach out to individuals. This was preferable to signposting, which placed an onus on the individual to seek further help outside of the immediate environment in which they were looking.

#### Fora

Another common suggestion was that help sites could facilitate a sense of community and interaction with peers via links to forums. This stemmed from a sense that ‘true’ understanding can only be gained from those who have ‘been through it’ (SH15). A small number of participants described benefitting from such a link and the immediacy this provided. However, discussion recurred about the need for a professional presence within such spaces to provide support and avoid suicide glorification, descriptions of suicidal feelings or behaviour that may trigger others, and trolling. Participants believed this could be fulfilled if affiliated with formal help providers and mental health charities. Three participants reinforced these arguments with exemplars of well-moderated forums they had accessed via a formal help site.

#### Lived-experience content

Access to recovery stories or other lived-experience dialogue was discussed as a specific form of content formal help pages could usefully incorporate, either through forums, video links, or static content. This could offer a unique type of ‘help’ otherwise absent from most formal help sites—hope, inspiration to get better, personal connection—and thus ‘a hook’ to retain users.

If there could be a link to survivor forums to pop up that would be a real big advantage. Hopefully, that would potentially put it out there for someone that before you consider suicide, look at these people that have beat it… it’s almost like, ‘here’s where you need to go for help, but here’s where you need to go for inspiration’... that would have helped me at the time, if I could have read, straight away, positive stories or support (SM107)

It [site] has to be structured in a way that within seconds you’re getting something positive… it would have to be an absorbing story, something that grabs my imagination… it’s about the voice in my head reading a story that drowns out everything else so it would have to be very emotive. (SM112).

Two participants praised specific formal sites for providing such content.

#### Self-help tools

Many (especially younger participants) desired links to ‘hands on’, interactive self-help tools with responsive ‘tips’ for managing feelings.

Reading an article on [depression] is actually quite tiresome. So maybe have a mood diary… then if it had advice relating to what you’d put into your mood diary… it could sort of monitor what triggers you were continually putting down then it would give you an idea of maybe go do this. (YP1)

Several had derived benefit from such tools and argued they should be more accessible. Examples included crisis planning tools, and the mood monitoring app ‘Moodscope’.

#### Signposting

A small number of participants emphasised the importance of signposting, indicating it was ‘reassuring’ (SM35) to know of available external help sources. Several had gone on to link with services. One (SM91) thought signposting was particularly valuable because it linked individuals to ‘the real world’ and so ‘did not encourage isolation’ unlike most of the internet content they had viewed.

## Discussion

This study provides insights into the use of online help provided by charities and healthcare organisation from a wide range of individuals experiencing suicidal feelings and behaviour. All but 2 of the 53 participants had come across such help, even though many did not search for it, indicating its accessibility. Engagement varied across levels of distress, participants being less likely to engage where suicidal thoughts were most severe. Though highly valued as a resource for information and advice for mental health problems, differing needs were expressed in the context of suicidal feelings, which existing sites did not appear to meet. Sites were then criticised as impersonal, dispassionate, too focused on information-giving, and for a tendency to suggest ‘tired’ or insensitive solutions incongruous with the reasons why some individuals go online for help, such as social isolation, fear of stigma, or reluctance to speak face-to-face. A primary emphasis on signposting was particularly criticised. Participants instead desired personal connection, actionable strategies, novel solutions (since many sought online help to supplement/replace ‘real-world’ help), and an immediate, in situ response. They suggested sites should incorporate interactive elements such as live chat, links to forums and self-help tools, and lived-experience content. Positive experiences of formal online help were consistent with these findings. This is the first time such views have been captured and can be used to suggest improvements to online resources.

Provision of help resources and emotional support is one way the internet can assist with suicide prevention [10]. Typically, help-seeking for suicidal feelings and behaviour is low, particularly amongst young people [11, 12]. The internet may offer a way to reach those reluctant to present to services [13]. Indeed, some studies find increased suicidality is linked to online help-seeking but offline help avoidance [14, 15].

Efforts have been made by Internet Service Providers to adjust search algorithms to prioritise help sites in suicide-related search output. However, research has focused on quantifying characteristics of help-seekers, trialling specific online interventions (e.g. apps), or examining online support groups, rather than seeking user evaluations of everyday online help pages. One small-scale survey [16] asked self-injuring adolescents (mostly female) about their preferences for online help. Data were limited to fixed-choice categories and one free-text question, and only a third of the sample had actually accessed online help. However, findings resonate with issues explored in-depth in our study. A preference for instant messaging, direct contact with a professional, forums and self-help programmes were the most commonly endorsed preferred sources of support. Furthermore, free-text comments expressed a desire for community, access to shared experience, and contact with a ‘real person’. Less than 20% of participants wanted information about self-harm and even fewer indicated a wish for signposting; a third reported they would only want online help and over 50% that they would want online help initially.

### Strengths and limitations

We employed robust qualitative methods to engage first-hand and in-depth with distressed/suicidal internet users in an area more often explored using survey methods (e.g. [17, 18]). Participants were recruited from three differing sources, thus increasing diversity and the transferability of findings, and resulting in a substantial dataset of over 50 interviews. The study included individuals with differing levels of suicidality, from the general population and from hospital settings, and reporting varying levels of service use and online behaviours. Sample SM was recruited through the online networks of a suicide prevention charity maximising likely representation of individuals using the internet for help-seeking. Positive accounts of formal online help appeared as unusual (‘deviant’) cases and provided an analytical tool for delineating characteristics of preferred online help.

Recruitment in studies of self-harm is known to be difficult [19] and in this study was most challenging in the young people sample, however, purposive sampling allowed us to over-sample hard to reach groups (young men), to ensure their inclusion. While most participants had viewed formal online help pages, relatively few had used these regularly or to any great extent. Valuable insights from those who described positive encounters with online help indicate that to gain a more rounded picture, further research could usefully target and explore the characteristics of individuals who are engaged by such provision. Also, many of the participants described a history of mental health difficulties. Individuals experiencing crisis for the first time may have found the information contained online more novel and useful.

The study was conducted in England, which is likely to have skewed the online resources viewed, though international help services are easily accessible through search engines and links and were also discussed by some participants. Some developments in online help provision have also occurred since these data were collected. Nevertheless, the findings highlight key features of provision deemed helpful and unhelpful and, therefore, can serve as a guide for help providers for onwards development.

### Implications

The Internet is commonly consulted by those experiencing suicidal thoughts. Formal online suicide help is readily accessible and there is high exposure to this, even amongst higher severity users who tend not to seek this. However, help provision only engaged and met the preferences of a small group of participants. Our findings, therefore, suggest that at present, this opportunity for suicide prevention is not being fully realised. Moreover, shortcomings of online help provision had on occasion resulted in a sense of hopelessness which, in turn, triggered negative online behaviour such as searching for suicide methods information. Content and engagement strategies both need to be addressed, recognising that the needs of an individual experiencing suicidal thoughts or crisis are different from those looking for general mental health help provision.

Online help provision needs to present a novel alternative to offline services—not simply a rolling out of or signposting to them—and to be tailored to the reasons why some people opt for virtual rather than ‘real world help’. Individuals require a direct response or solution within the environment in which they are searching and a focus on crisis management. However, it is noteworthy that some participants found signposting information helpful, indicating this should be included within the spectrum of responses. The finding that individuals were more likely to engage with online help prior to and post—not during—crisis suggests an urgent need to develop online help that can interrupt, engage and help severe users with higher suicidal intent and/or in active crisis. Participants’ visions for improvement suggest limitations may be addressed by capitalising upon unique features of the internet such as the capacity for dialogue, community, personal (yet anonymous) exchange, and sharing of experiential knowledge.

However, participants’ suggestions are not without difficulty. Linking to forums and lived-experience material requires careful moderation and crafting since in some circumstances these can have detrimental effects, leading to suicide contagion or incitement [7]. A recovery story, for instance, can be either helpful or harmful to a distressed individual depending on the focus and content of its narrative [20]. While formal help providers could serve as gatekeepers to safe interactive spaces, this would have significant resource and cost implications, as would a ‘live chat’ facility. This is pertinent since most formal help providers are charitable organisations.

Participants’ negative evaluations should also be placed in context, acknowledging (as a few participants did in retrospect) that some of their criticisms stemmed in part from the fact they were searching for the unobtainable and intangible—the internet being assigned a role of great panacea. Delivering a unique and immediate solution may not be attainable, however, participants’ evaluations do provide important insights for shaping the future of online suicide help provision.

## Conclusions and future directions

This study has made a case for an overhaul of online help services to ensure these meet users’ needs and more effectively capitalise upon the opportunity available for suicide prevention. This should be informed by further research investigating when and why distressed users access online help, their needs and expectations, and how they can be engaged. Importantly, research should explore possible approaches to retaining individuals with high suicidal intent who may initially ‘stumble across’ rather than deliberately access help. The online world is constantly evolving and there have been recent developments in suicide help provision. In the UK, limited opportunities for live chat are now available, Samaritans (the leading suicide prevention charity) are repackaging their web-based services, the text service SHOUT (https://www.giveusashout.org) launched in 2019, and a new suicide prevention website—StayingSafe.net—was launched in November 2018 following consultation with people with lived-experience and includes strategies for developing a safety plan and lived-experience video footage. It is, therefore, important to evaluate recent developments and identify models of effective help provision. Users should be involved not only in such evaluation, but also as co-producers of new online materials and approaches.

## References

[1] Mok K, Jorm A, Pirkis J (2016). Who goes online for suicide-related reasons? A comparison of suicidal people who use the internet for suicide-related reasons and those who do not. Crisis.

[2] Mars B, Heron J, Biddle L, Donovan J, Holley R, Piper M, Potokar J, Willey C, Gunnell D (2015). Exposure to, and searching for, information about suicide and self-harm on the Internet: Prevalence and predictors in a population based cohort of young adults. J Affect Disord.

[3] Padmanthan P, Biddle L, Carroll R, Derges J, Potokar J, Gunnell D (2018). Suicide and self-harm related internet use: a cross-sectional study and clinician focus groups. Crisis.

[4] Lai MH, Maniam T, Chan LF, Ravindran AV (2014). Caught in the web: a review of web-based suicide prevention. J Med Internet Res.

[5] Robinson J, Cox G, Bailey E, Hetrick S, Rodrigues M, Fisher S, Herrman H (2016). Social media and suicide prevention: a systematic review. Early Interv Psychiatry.

[6] Thornton L, Handley T, Kay-lambkin F, Baker A (2017). Is a person thinking about suicide likely to find help on the internet? An evaluation of Google search results. Suicide Life-Threat.

[7] Biddle L, Derges J, Goldsmith C, Donovan J, Gunnell D (2018). Using the internet for suicide-related purposes: contrasting findings from young people in the community and self-harm patients admitted to hospital. PLoS ONE.

[8] Boyd A, Golding J, MacLeod J, Lawlor D, Fraser A, Henderson J, Molloy L, Ness A, Ring S, Davey Smith G (2013). Cohort profile: the ‘Children of the 90s’—the index offspring of the Avon Longitudinal Study of Parents and Children. Int J Epidemiol.

[9] Glaser B, Strauss A (1967). The discovery of grounded theory: strategies for qualitative research.

[10] Luxton D, June J, Fairall J (2012). Social media and suicide: a public health perspective. Am J Public Health.

[11] Biddle L, Gunnell D, Sharp D, Donovan J (2004). Factors influencing help-seeking in mentally distressed young adults: a cross-sectional survey. Br J Gen Pract.

[12] Michelmore L, Hindley P (2012). Help-seeking for suicidal thoughts and self-harm in young people: a systematic review. Suicide Life Threat.

[13] Greidanus E, Everall R (2010). Helper therapy in an online suicide prevention community. Br J Guid Couns.

[14] Frost M, Casey L (2016). Who seeks help online for self-injury?. Arch Suicide Res.

[15] Seward A, Harris K (2016). Offline versus online suicide-related help seeking: changing domains, changing paradigms. J Clin Psychol.

[16] Frost M, Casey L, Rando N (2016). Self-injury, help-seeking and the internet: informing online service provision for young people. Crisis.

[17] Harris K, McLean J, Sheffield J (2009). Examining suicide-risk individuals who go online for suicide-related purposes. Arch Suicide Res.

[18] Bell J, Mok K, Gardiner E, Pirkis J (2018). Suicide-related internet use among suicidal young people in the UK: characteristics of users, effects if use, and barriers to offline help-seeking. Arch Suicide Res.

[19] Hawton K, Sinclair J (2003). The challenge of evaluating the effectiveness of treatments for deliberate self-harm. Psychol Med.

[20] Niederkrotenthaler T, Voracek M, Herberth A, Till B (2010). Role of media reports in completed and prevented suicide: Werther v. Papageno effects. Br J Psychiatry.

